# The Role of Social and Ability Belonging in Men’s and Women’s pSTEM Persistence

**DOI:** 10.3389/fpsyg.2019.02386

**Published:** 2019-10-31

**Authors:** Sarah Banchefsky, Karyn L. Lewis, Tiffany A. Ito

**Affiliations:** ^1^Office of Data Analytics, University of Colorado Boulder, Boulder, CO, United States; ^2^Center for School and Student Progress, NWEA, Portland, OR, United States; ^3^Department of Psychology and Neuroscience, University of Colorado Boulder, Boulder, CO, United States

**Keywords:** pSTEM, STEM, gender, social belonging, ability belonging, persistence, self-efficacy, identification

## Abstract

The benefits of belonging for academic performance and persistence have been examined primarily in terms of subjective perceptions of social belonging, but feeling ability belonging, or fit with one’s peers intellectually, is likely also important for academic success. This may particularly be the case in male-dominated fields, where inherent genius and natural talent are viewed as prerequisites for success. We tested the hypothesis that social and ability belonging each explain intentions to persist in physical science, technology, engineering, and math (pSTEM). We further explore whether women experience lower social and ability belonging than men on average in pSTEM and whether belonging more strongly relates to intentions to persist for women. At three time points throughout a semester, we assessed undergraduate pSTEM majors enrolled in a foundational calculus or physics course. Women reported lower pSTEM ability belonging and self-efficacy than men but higher identification with pSTEM. End-of-semester social belonging, ability belonging, and identification predicted intentions to persist in pSTEM, with a stronger relationship between social belonging and intentions to persist in pSTEM for women than men. These findings held after controlling for prior and current academic performance, as well as two conventional psychological predictors of academic success.

## Introduction

“It’s important to appreciate the background of endless skepticism that every woman in tech faces, and the resulting exhaustion we feel as the legitimacy of our presence is constantly questioned…There is always a jury, and it’s always still out.” –Cynthia Lee, Ph.D.

Women remain starkly underrepresented in the physical sciences, technology, engineering, and mathematics (pSTEM) in many countries, including the United States, fields offering careers that are lucrative (Chamberlain and Jayaraman, 2017) and plentiful (President’s Council of Advisors on Science and Technology [PCAST], 2012). At the university level in the U.S. in 2012, women obtained just 20% of engineering degrees, 19% of physics degrees, and 18% of computer science degrees (National Science Foundation and National Center for Science and Engineering Statistics, 2015). Moreover, roughly 35% of United States students, despite being well qualified and adequately prepared in math and science, abandon pSTEM fields during the first few semesters of college (Seymour and Hewitt, 1997; Daempfle, 2003); this percentage is even higher among women (Seymour and Hewitt, 1997; Huang et al., 2000; Blickenstaff, 2005; Vogt et al., 2007; Ohland et al., 2008; Chen, 2013; Ellis et al., 2016). If merely 10% of students who leave STEM majors during higher education could be retained, the United States could achieve its future national workforce needs, which are currently deficient in STEM fields (President’s Council of Advisors on Science and Technology [PCAST], 2012).

A number of social and interpersonal factors underlie the gender gap in representation within pSTEM fields, ranging from personal life choices—constrained or freely made—to unwelcoming masculine cultures (for reviews, see Eccles, 1994; Blickenstaff, 2005; Ceci et al., 2009; Cheryan et al., 2017). Of these many factors, recent research suggests a large role for social belonging (Lewis et al., 2016), which entails feeling like a valued, accepted, and legitimate member of a particular environment (Goodenow, 1993; Baumeister and Leary, 1995).^1^ The need to belong and form interpersonal attachments with others is a fundamental, ubiquitous human motivation related to both physical and psychological health (Baumeister and Leary, 1995; Cacioppo and Cacioppo, 2014).

Not surprisingly, people who anticipate belonging in pSTEM fields express greater interest in pursuing such fields in the first place (Murphy et al., 2007; Cheryan and Plaut, 2010). Once pursuing a pSTEM education, social belonging is related to persistence (Goodenow, 1993; Freeman et al., 2007; Walton and Cohen, 2007; Hausmann et al., 2009; Walton and Cohen, 2011; Good et al., 2012; Thoman et al., 2014; Eddy and Brownell, 2016) even after accounting for objective ability and confidence that one can successfully complete the tasks required for success (i.e., self-efficacy; Bandura, 1977; Wilson et al., 2015; Lewis et al., 2017).

Schmader and Sedikides (2018) recently argued that sense of “fit” with an environment is multifaceted and dimensions other than social fit also influence an individual’s decision to pursue a domain (see also Höhne and Zander, 2019). They also theorize that evaluations of social belonging are relevant only when social interactions are expected. Interacting with instructors and peers is an inherent component of pursuing a course of study. However, there are likely other relevant considerations, including how well one’s aptitude meets the perceived requirements of the domain (McPherson et al., 2018b). Consistent with this, Lewis and Hodges (2015) found that the degree of intellectual fit—the subjective sense that one possesses the same abilities, skills, and knowledge as one’s peers—predicts motivations and intentions to persist academically among undergraduates enrolled in psychology and linguistics courses (note that they refer to low levels of ability belonging as “ability belonging uncertainty”). pSTEM students were not examined in this past research, but the heavy emphasis within many pSTEM fields on inherent genius and natural talent is well documented (Leslie et al., 2015; Meyer et al., 2015; Ito and McPherson, 2018; Deiglmayr et al., 2019) and likely makes intellectual fit particularly relevant within pSTEM.

Given the important role of belonging in predicting persistence, it is critical to consider not only whether there are gender disparities in pSTEM belonging, and whether these disparities help explain the dearth of women in male-dominated fields, but also in what specific dimensions of belonging gender differences occur. Research indicates that women experience lower social belonging than men in male-dominated fields such as physics and computing (Lewis et al., 2016, 2017). Women’s lower social belonging is likely due to a number of factors unique to male-dominated fields: the dearth of women (Murphy et al., 2007; Dasgupta et al., 2015), the lack of relatable role models (Cheryan et al., 2013), subtly unwelcoming or even overtly hostile masculine cultures (Settles et al., 2006; Cheryan et al., 2017), the greater prevalence of sexist jokes (Gonsalves et al., 2016), and non-verbal behavior from men that excludes women from professional conversations (Barthelemy et al., 2016).

The same cues that erode women’s sense of social belonging likely also erode their sense of ability belonging—their belief that they have the same abilities and intellectual capacity as their peers. For example, Smith et al. (2013) found that compared to men, women in STEM graduate programs believed they worked harder than the average student in order to achieve the same outcome. In a second study, they demonstrated that the male-dominated status of a given field drove women’s concerns about working harder for the same results. Specifically, undergraduates considered a fictional “eco-psychology” graduate program. When eco-psychology was depicted as male-dominated rather than gender-balanced, women anticipated working harder than the typical student to achieve success, which in turn diminished their interest in pursuing the program (Smith et al., 2013).

As far as we are aware, whether women in fact experience lower ability belonging than men in pSTEM fields has not been specifically examined, although women recruited from psychology and linguistics classes did express lower ability belonging than men in two of three studies, even after accounting for their objective ability (GPAs; Lewis and Hodges, 2015). If anything, we suspect that this existing gender gap in ability belonging will be exacerbated in male-dominated fields, where (1) natural ability is valued and viewed as necessary for success (Leslie et al., 2015) and (2) women’s natural ability is negatively stereotyped (Spencer et al., 1999; Tiedemann, 2000; Stephens-Davidowitz, 2014). Together, these factors may make ability belonging a particularly relevant consideration in women’s decisions about whether or not to persist in pSTEM fields.

In addition to mean gender differences in social and ability belonging, both facets of belonging may be more important factors for women’s progression in pSTEM fields than men’s. The vulnerability hypothesis (Hughes et al., 2015) states that the individuals most at risk of failure in a particular academic setting will be most affected by their subjective experiences within that setting (see also Johnson et al., 2007; Walton and Cohen, 2007, 2011; Murphy and Zirkel, 2015). Supporting this hypothesis, multiple studies show that social belonging is a stronger predictor of academic outcomes among historically marginalized and negatively stereotyped groups, such as women in male-dominated pSTEM fields (Holleran et al., 2011; Good et al., 2012; Walton et al., 2015). Indeed, recent research found not only that women report lower average social belonging than men in computing and physics but also that social belonging more strongly predicted persistence in their major for women than men (Lewis et al., 2017).

Although a small body of research indicates that social belonging is more important to women’s persistence than men’s in male-dominated majors, it remains to be tested whether ability belonging is likewise a stronger predictor of women’s persistence. Just as women may be more prone to scan their environment and daily experiences for examples of whether they socially belong (Cheryan et al., 2009), they may also be more attuned to experiences confirming or denying whether they intellectually belong. In addition, recent research shows that female pSTEM majors more strongly believe that their fields require innate brilliance than their male peers do (Deiglmayr et al., 2019). This greater expectation that brilliance is required may make women more sensitive to their subjective assessments of ability fit than men.

## Current Research

Expanding upon prior research on belonging and in keeping with recent theorizing on the importance of different aspects of fit (Lewis and Hodges, 2015; Schmader and Sedikides, 2018), the current work examines gender differences in social and ability belonging, as well as whether each type of belonging is more tightly tied to women’s intentions to persist in pSTEM than men’s. We provide a stringent test of the relationship between these variables by accounting for objective academic performance as well as other theoretically important constructs linked to persistence, namely, self-efficacy and identification. In a recent review of possible psychological variables underlying gender disparities in STEM fields, Eddy and Brownell (2016) pointed to (social) belonging, self-efficacy, and identification as three key factors. Together, we call the four variables we measured—social belonging, ability belonging, self-efficacy, and identification—academic self-perceptions (ASPs).

Academic self-efficacy is the belief that one is capable of succeeding in specific academic tasks and goals such as exams and coursework (Bandura, 1977; Multon et al., 1991; Usher and Pajares, 2008). Robustly related to student performance, motivation, and persistence (Lent et al., 1986; Multon et al., 1991), women’s lower self-efficacy compared to men is frequently cited as underlying the lack of women in pSTEM fields (Besterfield-Sacre et al., 2001; Stout et al., 2011; Tellhed et al., 2017). Even when objective performance is identical, women tend to report lower self-efficacy than men in pSTEM fields (Correll, 2001, 2004; Spelke, 2005; Else-Quest et al., 2010; Sikora and Pokropek, 2012).

Although correlated, it is worth noting that ability belonging is distinct from self-efficacy (Lewis and Hodges, 2015). Self-efficacy captures confidence that one can succeed in tasks, and does not entail direct social comparison to one’s peers. A student could have high self-efficacy (e.g., believe she can perform well on exams) but nevertheless believe she has lower intellectual aptitude than her peers. In contrast, a student could theoretically have low self-efficacy and question his ability to perform well on homework and exams but still believe he is as or more capable than his peers.

Academic identification refers to caring about one’s performance in a given domain and basing one’s self-worth or self-esteem upon performance in that domain (Spencer et al., 1999; Chemers et al., 2011; Osborne and Jones, 2011; Cundiff et al., 2013). Students highly identified with pSTEM view it as an important aspect of “who they are,” an identity from which they draw meaning and self-esteem. Research shows that in pSTEM fields, identification is related to positive academic outcomes including higher performance, motivation, and commitment to doing well (Smith and White, 2001), as well as greater likelihood of choosing a pSTEM career (Hazari et al., 2010). The latter two studies also found that men expressed stronger pSTEM identification than women, suggesting that there may also be gender differences favoring men regarding pSTEM identification.

In summary, research has shown a clear relationship between self-efficacy (e.g., I can succeed in this field), identification (e.g., this field is important to me), and positive outcomes in a field. In addition to these factors, we maintain that belonging—as a basic human need and motivation—should be tied to intentions to persist in a given field, over and above self-efficacy and identification (Lewis et al., 2017; Ito and McPherson, 2018). Specifically, we predicted that over and above prior and current academic performance, women would report lower ability and social belonging in pSTEM than men on average across the semester (Hypothesis 1); that both ability and social belonging would be related to intentions to persist among both women and men even after accounting for self-efficacy and identification (Hypothesis 2); and that the relationship between social and ability belonging and pSTEM persistence would be stronger for women than for men (Hypothesis 3).

## Materials and Methods

### Participants

Participants were undergraduates taking a large gateway physics or calculus course, both of which are required to advance in nearly all pSTEM majors at the university where data were collected. There were three physics sections, all taught by the same male professor, and seven calculus sections, two of which were taught by the same male professor and five of which were each taught by a different male professor. More information on the courses is available in Section 1 in the Supplementary Material.

Selecting from introductory courses ensured obtaining a sample of students early in their academic path, when attrition is highest (Daempfle, 2003). Introductory physics and calculus courses were selected in particular because women’s representation in physics is among the lowest of any pSTEM field (National Science Foundation and National Center for Science and Engineering Statistics, 2015), and women are 1.5 times more likely to leave the pSTEM pipeline after college calculus compared to men (Ellis et al., 2016), making it particularly relevant to understand gender disparities among students in these classes. Both courses are historically male-dominated (in our sample, 21.24% of physics students and 25.81% of calculus students were women), consist primarily of students majoring in pSTEM, and are the first in a sequence of required courses for pSTEM majors at the university (for example, both courses are required for all engineering majors, who comprised the majority of our sample).

All students enrolled in the selected physics (*n* = 831) or calculus (*n* = 648) sections at the beginning of the fall semester were contacted via university email and invited to participate in the study in exchange for compensation (see Procedures for details). Of the 1,479 contacted, 599 responded at Time 1 (40.50%). After data collection, three exclusion criteria were applied. First, because we were interested in the persistence of students pursuing pSTEM fields, students who either self-reported being a non-pSTEM major or were undecided about their major (*n* = 30) were removed (see Section 2 in the Supplementary Material, for qualifying pSTEM majors). Second, we removed students enrolled in honors sections of the courses (*n* = 31) because these courses were structured differently and had much smaller enrollments relative to the large-lecture courses. Third, students whose institutional records indicated that they withdrew from the course or received an “incomplete” (*n* = 29) were removed because (1) most did not complete the third survey and were therefore missing data on intentions to persist in pSTEM and (2) intentions to persist in pSTEM fields are inherently constrained for students who have not completed a required gateway pSTEM course. Given that the three exclusion criteria could overlap, the final sample of eligible participants included 516 students.

Of these, 121 (23%) self-identified as women (21.07% of the physics sample and 26.27% of the calculus sample). A majority of students (84.30%) were engineering majors, followed by physics (6.59%), astronomy (2.71%), chemistry (2.32%), biochemistry (1.74%), and mathematics (1.36%). The majority identified as White (70.54%), followed by Asian (9.50%), other (9.30%; the majority wrote in “Asian,” but there was also “Indian,” “Middle Eastern,” and “Arab”), Hispanic (3.68%), and Black (0.39%); 6.20% selected more than one racial category. Institutional data (available for *n* = 398 or 77.13% of students who permitted access) indicated that the sample was primarily first-year students (90.70%), followed by sophomores (7.79%), juniors (1.26%), and seniors (0.25%).

This study was carried out in accordance with the recommendations of United States Office for Human Research Protections. The protocol was approved by the University of Colorado’s Institutional Review Board. All subjects gave written informed consent in accordance with the Declaration of Helsinki.

### Materials

These data are part of a broader study that encompassed three surveys and a variety of other measures beyond the focus of this paper (documented in Section 3 in the Supplementary Material). The current study focuses on a subset of the measures (all of which can be viewed in Table 1) and primarily on Surveys 1 and 3, conducted at the beginning and end of the semester. The response format for items was a 5-point Likert scale (1 = strongly disagree, 5 = strongly agree). All scales demonstrated high reliability (see Table 2).

**TABLE 1:** Academic self-perceptions and intentions to persist in STEM.

**Construct**	**Items**
pSTEM social belonging	I feel like I belong in STEM.
	People in STEM accept me.
	I feel like an outsider in STEM. (r)
	I feel a connection with the STEM community.
	People in STEM are a lot like me.
	I fit in well in STEM.
pSTEM ability belonging	I sometimes feel like other students in STEM have skills that I don’t have. (r)
	I’m not sure that I’m cut out for STEM. (r)
	I feel similar to the kinds of people who have what it takes to succeed in STEM.
	I’m not certain I fit in intellectually in STEM. (r)
pSTEM identification	It is important to me that I am good at math and science.
	Doing well on math and science tests is important to my self-esteem.
	Is it important to me to perform well on science and math tests.
	Having strong math and science skills is important to me.
pSTEM self-efficacy	I am confident I can…
	Complete homework assignments by myself.
	Perform well on exams.
	Demonstrate what I know on exams.
	Learn STEM concepts.
	Complete the course with a B or better.
Intentions to persist in pSTEM	I could see myself going into a career related to STEM.
	I look forward to taking more STEM courses.
	It is my intention to major in a STEM discipline.
	I have no doubt that I will graduate with a degree in a STEM field.
	I have seriously considered changing my major to a non-STEM related field. (r)
	STEM is the right career path for me.

*pSTEM, physical sciences, technology, engineering, and mathematics. (r) Indicates reverse-scored.*

**TABLE 2:** Descriptive statistics for academic self-perceptions and pSTEM intentions.

**Construct**	**# Items**	**Mean alpha**	**Mean (Standard Deviation)**						**Gender difference**	
				
			**Overall**		**Men**		**Women**		***z*-value**	**Cohen’s *d***
Social belonging	6	0.85	3.82	(0.51)	3.81	(0.49)	3.83	(0.54)	0.40	0.04
Ability belonging	4	0.71	3.64	(0.55)	3.68	(0.53)	3.52	(0.59)	–2.76^∗∗^	0.29
Identification	4	0.78	4.37	(0.46)	4.34	(0.47)	4.44	(0.44)	2.14^∗^	0.21
Self-efficacy	5	0.88	4.12	(0.49)	4.19	(0.47)	3.91	(0.50)	–5.47^∗∗∗^	0.58
pSTEM intentions	6	0.90	4.12	(0.72)	4.13	(0.71)	4.06	(0.76)	–0.87	0.10

*^∗∗∗^*p* < 0.001, ^∗∗^*p* < 0.01, ^∗^*p* < 0.05. The scales ranged from 1 to 5. Alpha is the average of the separate alphas for each scale at Time 1, Time 2, and Time 3. The mean and standard deviation for the academic self-perceptions were grand means across the separate Time 1, Time 2, and Time 3 mean scores (e.g., the mean of belonging at Time 1, 2, and 3). Cohen’s *d* was also computed from the estimated means and standard deviations from the path model using full information maximum likelihood (FIML).*

#### Demographics

Self-reported demographics included gender (male, female, other), age, ethnicity (Black/African American, Asian American, Hispanic/Mexican American, White/Caucasian, and other), and academic major (open response).

#### Academic Self-Perceptions

At each time point, six items assessed social belonging (e.g., “I feel like I belong in STEM”; adapted from Walton and Cohen, 2007; Good et al., 2012; Lewis et al., 2016); four assessed ability belonging [e.g., “I’m not sure that I’m cut out for STEM” (reverse-scored); Lewis and Hodges, 2015]; four assessed identification (e.g., “It is important to me that I am good at STEM”; Spencer et al., 1999); and five assessed self-efficacy (e.g., “In STEM, I am confident that I can demonstrate what I know on exams”; Bandura, 1977; Betz and Hackett, 1983). For brevity in the survey, we did not specify that we were referring to physical sciences, but students in our sample were specifically majoring in physical sciences and not social sciences. We therefore refer to pSTEM throughout this paper. Given that two constructs refer to aspects of belonging, and that past operationalizations of social belonging include items that might be affected by perceptions of ability (e.g., “I feel like I belong in STEM”), we conducted comparative confirmatory factor analyses to assess whether ability and social belonging should be combined into one factor. Compared to the single-factor belonging model, fit was significantly improved when social and ability belonging were treated as separate factors. This was true at Time 1, χ^2^ difference (1) = 55.74, Time 2, χ^2^ difference (1) = 75.64, and Time 3, χ^2^ difference (1) = 59.22, all *p*s < 0.001.

#### pSTEM Intentions

The key dependent variable was self-reported intentions to persist in pSTEM (see Table 1), assessed by six items in the Time 3 survey near the end of the semester (e.g., “It is my intention to major in a STEM discipline”; α = 0.90). Intentions are a proximal predictor of behavior (Ajzen, 1985, 1987, 1991, 2011) and consequently a frequently used measure of educational outcomes (e.g., Tinto, 1997; Murphy et al., 2007; Good et al., 2012; Lewis et al., 2017; Ito and McPherson, 2018). Studies also show a strong association between academic intentions to persist and actual persistence (e.g., Bean, 1982; Voorhees, 1987; Cabrera et al., 1993; Davidson et al., 2009; Luke et al., 2015).

#### Prior and Current Academic Performance

To account for prior academic performance, we accounted for high-school GPA and scores on standardized entrance exams (SAT math, ACT math, ACT science). Of participants who provided access to institutional records, SAT math scores were available for 164 (41.21%), ACT math and science-reasoning for 309 participants (77.64%), and 108 had records for both (27.13%). For each student, each available test score was standardized and, if appropriate, averaged into one index capturing standardized math/science test performance. To account for ongoing objective performance, we calculated the average GPA across all pSTEM courses during the semester in which the surveys were administered (including the course they were enrolled in; available for 98.99% of students who granted permission to access institutional records).

#### Missing Data

The 516 eligible participants who responded to the Time 1 survey (*n* = 280 in physics and *n* = 236 in calculus) were invited to participate in subsequent surveys. At Time 3, 441 participants responded (85.46% retained from initial enrollment). Women were marginally more likely to be retained at Time 3 than men [90.91% versus 83.80%, χ^2^(1) = 3.22, *p* = 0.07].

Little’s (1988) Missing Completely at Random (MCAR) test was performed in R to examine missing patterns in the data. The test included the following 15 variables included in the most complex models: intentions to persist in STEM, the four ASPs at Time 1 and Time 3, self-reported gender, course, and the four codes capturing course professor. Institutional record data were not included in the MCAR test because these were not missing at random—rather, participants had the option of giving us access to these records (77.13% did so). The test indicated that the data were not missing completely at random: there were five patterns of missing data, *p* < 0.01 (the null hypothesis being that the data are missing completely at random). This is unsurprising given the longitudinal nature of the data, and moreover, the use of full information maximum likelihood (FIML) estimation accounts for missing data, even if not missing completely at random (Baraldi and Enders, 2010).

### Procedures

Participants received an email invitation stating that we were interested in “issues that students in science and math majors at CU Boulder face” and that they were being contacted because they were enrolled in a science or math course. They were informed that their participation would consist of completing up to three online surveys regarding their experiences in their courses. Participants received $10 USD for completing the first survey, $15 USD for the second survey, and $20 USD for the final survey. To encourage complete participation, a $10 USD bonus was offered for completing all three surveys, and students were also entered into a raffle to win an additional prize (ranging from $25 to $50 USD).

The first survey was administered at the beginning of the semester (Time 1; Week 2 of the 16-week semester), the second was administered at the middle of the semester (Time 2; Week 8), and the final survey was administered at the end of the semester (Time 3; Week 16). Each survey was opened the day the invitations were emailed and remained open for 2 weeks. Reminder emails were sent to participants who had not yet completed the survey 1 week after it opened and 1 day before it closed. At the end of each survey, participants were then asked how they would like to receive their payment (Amazon gift card or cash pickup). When data collection was finalized, all participants were emailed a debriefing form.

At Time 1, participants gave informed consent, completed demographics, and were asked whether we could have their student identification number in order to access their institutional records. At all time points, participants first completed the ASP items in a fixed random order. Prior to the measures of interest here, participants were asked about experiences in their particular course (see Section 3 in the Supplementary Material). They were then informed, “The following questions ask about your experiences and perceptions of the broader field of science, engineering, and math (STEM) in general,” before completing the social belonging, ability belonging, and identification items intermixed in a fixed, random order. The self-efficacy items were presented on the next page and had a slightly different prompt: “Please rate how confident you are that you can do each of the following things.” Other items not relevant to the current manuscript were then completed (see Section 3 in the Supplementary Material). At Time 3 only, participants reported their intentions to persist in pSTEM after reporting their perceived course utility, followed by the additional measures in Section 3 in the Supplementary Material.

## Results

### Preliminary Analyses

**FIGURE 1:**
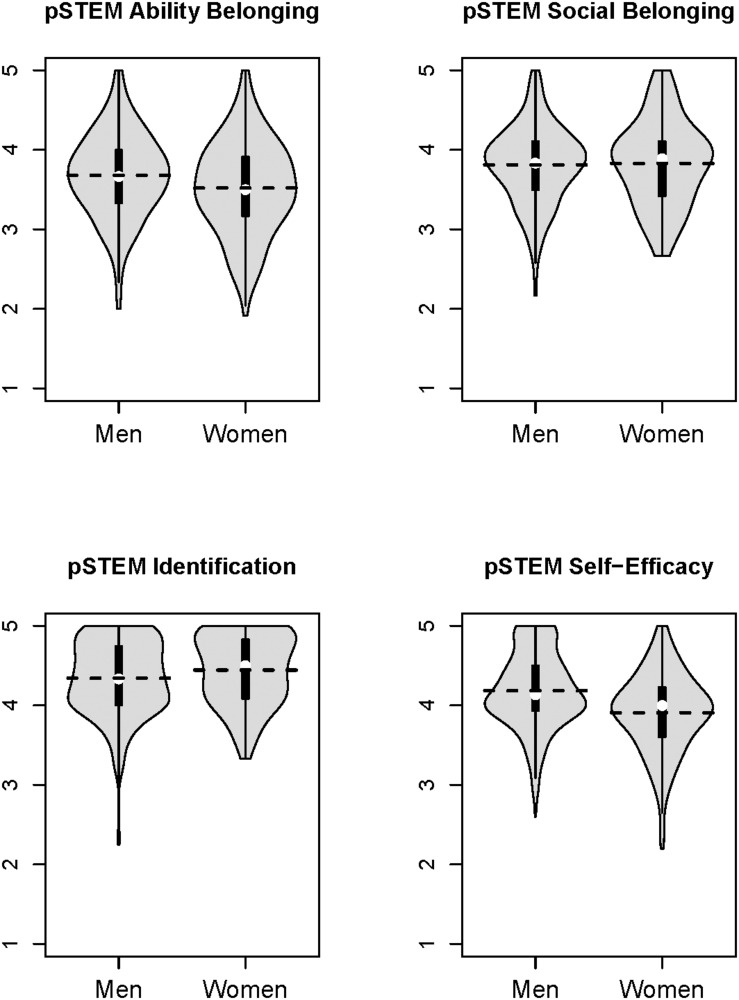
Violin plots depicting the academic self-perceptions on average over the semester for men and women. The boxplot and whiskers are in black; the white circle represents the median, and dashed horizontal lines represent the mean. The distribution of responses, in gray, is reflected on each side of the boxplot.

On average across the semester (averaging across Time 1, Time 2, and Time 3), students reported relatively high social and ability belonging, identification, and self-efficacy (see Table 2). Women reported lower average ability belonging in pSTEM than men. However, women and men did not differ in social belonging in pSTEM. Women also expressed lower average pSTEM self-efficacy, but greater average pSTEM identification, compared to men (see Table 2 and Figure 1). Students expressed strong intentions to persist in pSTEM, which did not differ by gender. Regarding prior performance, women had lower standardized math and science test scores than men, unstandardized *b* = −0.12, *z* = −2.02, *p* = 0.04, but there was no gender difference in high-school GPA, *b* = 0.04, *z* = 1.763, *p* = 0.10. Regarding ongoing academic performance, there was also no gender difference in pSTEM GPA that semester, *z* = −0.03, *p* = 0.45.

Table 3 depicts correlations controlling for participant gender. Overall, the ASPs were themselves highly positively correlated; students who felt greater social belonging also tended to have greater ability belonging, identification, and self-efficacy. Notably, the correlation between social and ability belonging was very similar to that observed in prior research (*r* = 0.63 here and *r* = 0.60 in Lewis and Hodges, 2015). Standardized test scores and high-school GPA were positively correlated with social belonging, ability belonging, and self-efficacy, but were unrelated to identification. Whereas standardized test scores in math and science were positively related to intentions to persist in pSTEM, high-school GPA was notably unrelated to pSTEM intentions. Finally, pSTEM GPA was positively related to all variables—prior performance, each ASP, as well as intentions to persist in pSTEM.

**TABLE 3:** Correlations and descriptive statistics of prior and ongoing performance, academic self-perceptions, and outcomes, partialing gender.

	**1**	**2**	**3**	**4**	**5**	**6**	**7**	**8**
(1) SAT/ACT math/science								
(2) High-school GPA	**0.32**							
(3) Average social belonging	**0.23**	**0.18**						
(4) Average ability belonging	**0.30**	**0.12**	**0.63**					
(5) Average identification	0.01	−0.04	**0.40**	**0.24**				
(6) Average self-efficacy	**0.28**	**0.09**	**0.51**	**0.57**	**0.44**			
(7) pSTEM GPA	**0.48**	**0.32**	**0.34**	**0.35**	**0.14**	**0.40**		
(8) Intentions to persist in pSTEM	**0.14**	−0.06	**0.55**	**0.49**	**0.44**	**0.46**	**0.31**	
Mean	0	3.78	3.82	3.60	4.39	4.05	3.02	4.10
*sd*	1	0.39	0.50	0.55	0.46	0.48	0.68	0.72

*All correlations greater than or equal to an absolute value of 0.10 (in bold) are significant at *p* < 0.05. SAT/ACT math scores are standardized. *sd*, standard deviation. Self-perceptions are averaged over all three time points.*

### Analytic Strategy

Analyses assessing our three hypotheses were conducted in R version 1.0.136, using path models conducted with the R package lavaan (Rosseel, 2012). All continuous predictors were centered around their mean, and all categorical predictors were contrast-coded. Primary analyses examined gender (women = 1, men = −1), controlling for prior academic performance (i.e., standardized test scores in math and science and high-school GPA) and the students’ course professor for the course they were responding about in the survey (five orthogonal contrast-codes, one of which compared physics to calculus). We accounted for indicators of pSTEM achievement to ensure that any observed relationships and gender differences were not due to differences in prior or ongoing pSTEM performance. The inclusion of strong covariates can reduce the likelihood that observed associations are due to unmeasured confounds. We controlled for professor to account for non-independence in the data, which is likely to occur with grouped data. Here, student responses about their pSTEM experiences and their specific course professor are surely influenced by their professor, and students with the same professor may have more similar responses to each other. Ignoring non-independence biases the results (Judd et al., 2017). We also controlled for students’ academic year (e.g., freshmen, sophomores; numerically coded and mean-centered) because initial exploratory analyses indicated that women had a higher class standing than men on average, *t*(417) = 3.64, *p* < 0.001. Specifically, men were more likely to be freshmen (92.77% of men versus 83.17% of women), less likely to be sophomores (6.92% of men versus 11.88% of women), and less likely to be juniors (0.31% of men versus 3.96% of women).

All models used FIML estimation, the preferred analytic method to implicitly handle missing data. FIML provides less biased parameter estimates even if data are not missing completely at random (Baraldi and Enders, 2010). Rather than deleting observations with missing data on predictor variables as would occur using ordinary least squares estimation, FIML estimates the values of the predictors based on the available data. Importantly, this approach improved our statistical power because it retained students who did not provide access to institutional record data (*n* = 118).

Data were collected at three time points; thus, they were longitudinal in nature and could have been examined using latent growth curve modeling (LGCM; Curran et al., 2010). Although LGCM is a powerful technique to analyze longitudinal data, structural equation modeling (of which LGCM is a specific type) requires a large sample size, especially when the model is complex (Wolf et al., 2013); Kline (2011) recommends at least 200 participants per group, and our sample contains only 121 women. Initial attempts to use an LGCM approach were not fruitful—in particular, the models for women demonstrated estimation problems (e.g., negative latent variances, failures to converge). To account for the longitudinal nature of the data and enhance statistical power, we included ASPs (e.g., social belonging) at Time 1 and Time 3 as predictors of pSTEM persistence (for a similar analytic approach, see Good et al., 2012). This model specification enabled us to ask how a change in belonging from baseline (measured at the beginning of the semester) is related to pSTEM persistence. (A complete examination of how each ASP changed throughout the semester is beyond the scope of this paper, but see Section 4 in the Supplementary Material and Supplementary Table 1, for an analysis of how each changed over the course of the semester. In sum, social and ability belonging remained flat over the course of the semester, and identification and self-efficacy both dropped. Notably, identification and self-efficacy were near ceiling at Time 1. This initial ceiling effect may have contributed to their decline over the course of the semester.)

Finally, because some of the predictors showed high inter-correlations (in particular, social and ability belonging; see Table 3), we tested for multicollinearity, which occurs when two or more predictors in a model are highly correlated and can cause statistical estimations to be unreliable (Wooldridge, 2013; Thompson et al., 2017). To do so, we examined the variance inflation factors (VIFs), the extent to which variation in the model is inflated by the presence of correlation among predictor variables (Salmerón Gómez et al., 2016). This was done in a model regressing intentions to persist in pSTEM onto all four ASPs at Time 1 and Time 3 (each mean-centered), high-school GPA (mean-centered), standardized test performance, pSTEM GPA (mean-centered), gender (contrast-coded), academic year (mean-centered), and professor (five orthogonal contrast-codes).

The VIF model revealed that social and ability belonging at Time 3 were redundant enough to warrant being either combined or tested in separate models (see Supplementary Table 2) (Wooldridge, 2013). As noted in the Section “Materials and Methods,” factor analyses showed better fit when social and ability belonging were treated as separate factors rather than being combined into a single factor. These results, in combination with prior theoretical work establishing that social and ability belonging are two distinct constructs (Lewis and Hodges, 2015), motivated our choice to treat these as separate factors tested in two separate models. Details on the VIF analyses are provided in Section 5 in the Supplementary Material.

### Do Women Report Lower Social and Ability Belonging Than Men? (Hypothesis 1)

Hypothesis 1 was assessed with path models that accounted for important covariates—academic year, professor, and prior and ongoing performance (i.e., high-school GPA, SAT/ACT math scores, and pSTEM GPA). As seen in Table 4, the raw gender differences presented in Table 2 and Figure 1 largely persisted when controlling for these four additional variables. We found partial support for Hypothesis 1, that women would have lower social and ability belonging than men: women expressed the *same* pSTEM social belonging as men, *p* = 0.34, but marginally lower pSTEM ability belonging, b = −0.05, *z*-value = −1.82, *p* = 0.068.

**TABLE 4:** Effect of gender and covariates on academic self-perceptions.

**Predictors**	**Unstandardized betas**			
**Outcomes**	**Ability**	**Social**	**Self-**	**Identification**
	**belonging**	**belonging**	**efficacy**	
Gender (+1 = women, −1 = men)	−0.05^+^	0.02	**−0.11^∗∗∗^**	**0.06^∗^**
Academic year	−0.12	−0.11	**−0.13^∗^**	−0.04
High-school GPA	−0.05	0.08	−0.10	−0.08
SAT and ACT math and science	**0.09^∗∗^**	0.03	**0.06^∗^**	−0.03
pSTEM GPA	**0.22^∗∗∗^**	**0.20^∗∗∗^**	**0.25^∗∗∗^**	**0.12^∗∗^**
Prof code 1 (physics vs. calculus)	0.00	0.00	0.01	0.01
Prof code 2	0.01	0.00	0.00	0.01
Prof code 3	0.00	0.01	0.00	−0.02
Prof code 4	0.06	0.03	0.02	0.03
Prof code 5	0.04	0.01	**−0.07^∗^**	0.01
*R*^2^	17.00%	13.30%	24.10%	4.70%

*^∗∗∗^*p* < 0.001, ^∗∗^*p* < 0.01, ^∗^*p* < 0.05, ^+^*p* < 0.10. Prof, Professor. The first professor code captures whether students were evaluating their physics or calculus class. The other four orthogonal contrast-codes control for which professor the students had for their course. All continuous predictors were mean-centered, and categorical predictors were orthogonally contrast-coded. Significant effects are in bold.*

Women also expressed lower pSTEM self-efficacy than men, *b* = −0.11, *z*-value = −4.69, *p* < 0.001, but higher pSTEM identification, *b* = 0.06, *z* = 2.50, *p* = 0.012. In summary, over and above academic preparation and current academic performance, women cared even more about their performance and knowledge in pSTEM than did men (i.e., women’s pSTEM identification was greater than men’s) but were simultaneously more concerned that they did not have what it takes to succeed in pSTEM both individually (i.e., women’s pSTEM self-efficacy was lower than men’s) and relative to their peers (i.e., women’s pSTEM ability belonging was marginally lower than men’s).

### Do Social and Ability Belonging Predict pSTEM Persistence? (Hypothesis 2)

Our second hypothesis was that social and ability belonging would predict intentions to persist in pSTEM, even after accounting for self-efficacy and identification, academic preparation, and ongoing academic performance.

We included both belonging at Time 1 and that at Time 3 as predictors of pSTEM persistence (for a similar analytic approach see Good et al., 2012). This model specification enabled us to ask how change in belonging from baseline (measured at the beginning of the semester) is related to pSTEM persistence.

Due to multicollinearity between social and ability belonging, each predictor was tested in a separate model, one for social and one for ability belonging. Results are depicted in Tables 5, 6, each of which shows three models: the first model included only the academic preparation variables, current academic performance, and course professor codes. The second model adds the belonging variable of interest (either social or ability belonging). The comparison between model 1 and model 2 reveals the contribution of belonging to predicting persistence. Finally, a third model adds identification and self-efficacy to provide an assessment of the degree to which belonging continues to predict persistence when other critical aspects of ASPs are included.

**TABLE 5:** Parameter estimates social belonging—intentions to persist models.

	**Unstandardized betas**		
**Predictors**	**Model 1**	**Model 2 add**	**Model 3 add**
	**no ASP**	**belonging**	**other ASP**
Gender (+1 = women, −1 = men)	0.02	−0.02	−0.01
Academic year	−0.17	−0.09	−0.06
High-school GPA	**−0.36^∗∗^**	**−0.37^∗∗∗^**	**−0.32^∗∗^**
SAT and ACT math and science	0.04	0.00	0.01
pSTEM GPA	**0.37^∗∗∗^**	**0.18^∗∗^**	**0.13^∗^**
Prof code 1 (physics vs. calculus)	0.01	0.01	0.00
Prof code 2	−0.02	−0.03	−0.03
Prof code 3	−0.02	−0.03	−0.02
Prof code 4	−0.01	−0.00	0.01
Prof code 5	−0.08	−0.06	−0.03
Social belonging (Time 3)	–	**0.58^∗∗∗^**	**0.45^∗∗∗^**
Social belonging (Time 1)	–	**0.14^∗^**	0.09
Identification (Time 3)	–	–	**0.31^∗∗∗^**
Identification (Time 1)	–	–	0.03
Self-efficacy (Time 3)	–	–	0.07
Self-efficacy (Time 1)	–	–	0.07
*R*^2^	15.60%	38.00%	44.50%

*^∗∗∗^*p* < 0.001, ^∗∗^*p* < 0.01, ^∗^*p* < 0.05. The first professor code captures whether the students were evaluating their physics or calculus class. The other four orthogonal contrast-codes control for which professor the students had for their course. ASP, academic self-perception. All continuous predictors were mean-centered, and categorical predictors were orthogonally contrast-coded. Significant effects are in bold.*

**TABLE 6:** Parameter estimates ability belonging—intentions to persist models.

	**Unstandardized betas**		
**Predictors**	**Model 1**	**Model 2 add**	**Model 3 add**
	**no ASP**	**belonging**	**other ASP**
Gender (+1 = women, −1 = men)	0.02	0.02	0.01
Academic year	−0.17	−0.14	−0.10
High-school GPA	**−0.36^∗∗^**	**−0.35^∗∗∗^**	**−0.29^∗∗^**
SAT and ACT math and science	0.04	−0.02	0.00
pSTEM GPA	**0.37^∗∗∗^**	**0.21^∗∗^**	**0.13^∗^**
Prof code 1 (physics vs. calculus)	0.01	0.01	0.00
Prof code 2	−0.02	−0.02	−0.02
Prof code 3	−0.02	−0.03	−0.02
Prof code 4	−0.01	0.00	0.00
Prof code 5	−0.08	−0.07	−0.03
Ability belonging (Time 3)	–	**0.42^∗∗∗^**	**0.34^∗∗∗^**
Ability belonging (Time 1)	–	0.11^+^	0.05
Identification (Time 3)	–	–	**0.39^∗∗∗^**
Identification (Time 1)	–	–	0.07
Self-efficacy (Time 3)	–	–	0.07
Self-efficacy (Time 1)	–	–	0.06
*R*^2^	15.60%	30.00%	41.80%

*^∗∗∗^*p* < 0.001, ^∗∗^*p* < 0.01, ^∗^*p* < 0.05, ^+^*p* < 0.10. The first professor code captures whether students were evaluating their physics or calculus class. The other four orthogonal contrast-codes control for which professor the students had for their course. All continuous predictors were mean-centered, and categorical predictors were orthogonally contrast-coded. Significant effects are in bold.*

Results supported Hypothesis 2: ability and social belonging at the end of the semester were each strongly related to intentions to persist in pSTEM, even after accounting for identification and self-efficacy, as well as prior and ongoing academic performance (see Tables 5, 6, respectively). As seen by comparing columns 1 and 2 in Tables 5, 6, including either social or ability belonging as a predictor in the models explained more than twice the variance in intentions to persist in pSTEM (*R*^2^ = 38.00% and *R*^2^ = 30.00%, respectively) relative to the model without belonging (*R*^2^ = 15.60%). Furthermore, social and ability belonging at Time 3 remained strongly related to intentions to persist after accounting for initial social and ability belonging, as well as end-of-semester self-efficacy (unrelated to intentions to persist) and identification (significantly and positively related to intentions to persist). In sum, changes in the sense of fit within pSTEM, whether socially and intellectually, were significantly related to intentions to persist in pSTEM. Students entered pSTEM with a certain level of belonging, and the change they experienced in pSTEM belonging over the course of the semester was linked to their intentions to persist in pSTEM. This occurred even after accounting for prior academic performance (high-school GPA and standardized test scores), ongoing academic performance (pSTEM GPA), and changes in self-efficacy and identification over the course of the semester.

For completeness, we also conducted a path model including both predictors. This model indicated that both ability and social belonging were significant predictors of intentions to persist in pSTEM (for more details, see Supplementary Table 3). This suggests that social and ability belonging each uniquely explains intentions to persist in pSTEM. In other words, each type of belonging—social and ability—was significantly related to intentions to persist in pSTEM after controlling for the other type of belonging.

### Do Ability and Social Belonging Relate to pSTEM Persistence More for Women Than Men? (Hypothesis 3)

**FIGURE 2:**
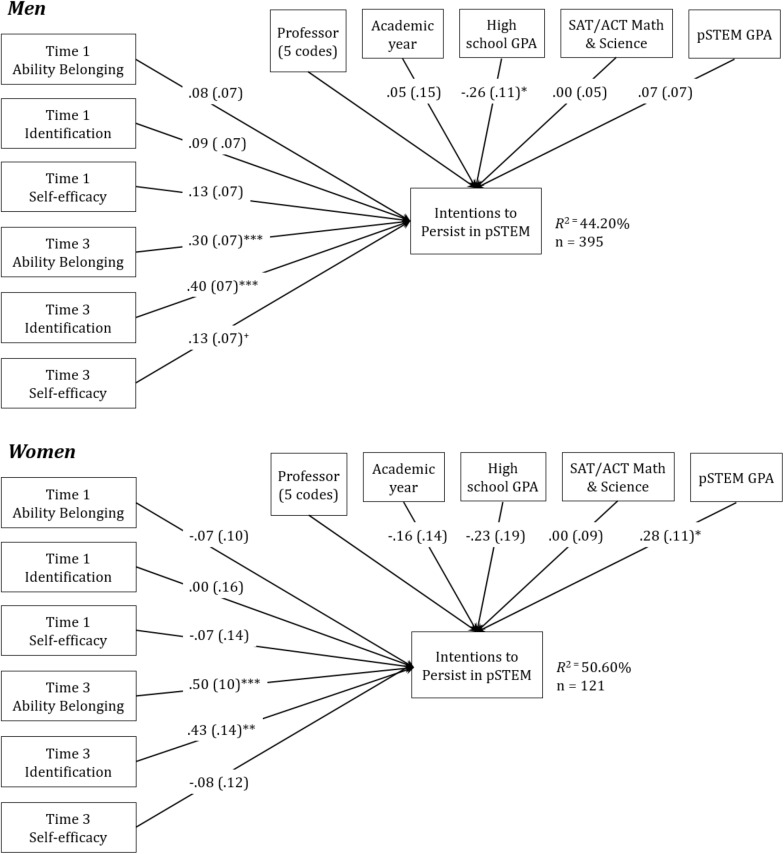
Path models depicting relationships among ability belonging and intentions to persist for men and women, controlling for professor, academic year, and prior and ongoing performance. Unstandardized betas are presented, with Standard Errors in parentheses. For simplicity, paths for professor are not depicted.

**FIGURE 3:**
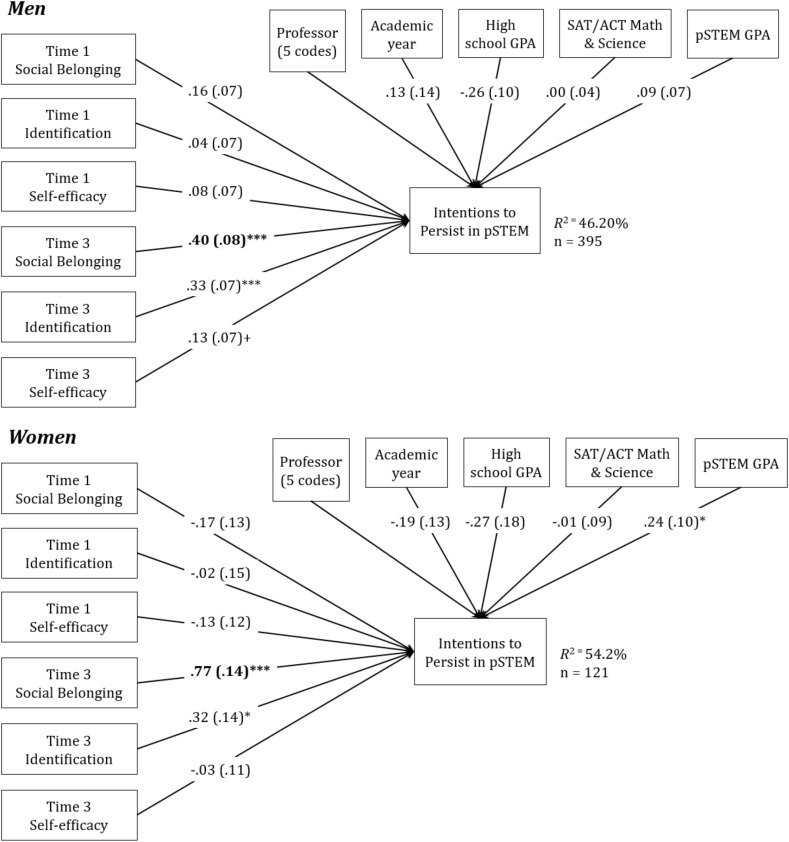
Path models depicting relationships among social belonging and intentions to persist for men and women, controlling for professor, academic year, and prior and ongoing performance. Unstandardized betas are presented, with Standard Errors in parentheses. Paths in bold were significantly different for women and men. For simplicity, paths for professor are not.

We next tested Hypothesis 3, that ability and social belonging would play a stronger role in women’s than men’s intentions to persist in pSTEM. To do so, gender invariance tests were conducted using the final models used to test Hypothesis 2, as shown in column 3 of Tables 5, 6. This entails comparing the chi-square of a model estimated separately for men and women to a model in which the path of interest (i.e., the relationship between end-of-semester social or ability belonging and intentions to persist in pSTEM) is constrained to be equivalent for the genders (see Figures 2, 3). If the constrained model results in significantly reduced goodness of fit, the tested path is significantly different for women and men.

As seen in Figure 2, the relationship between end-of-semester ability belonging and intentions to persist was directionally 1.67 times stronger for women [beta (*b*) = 0.50, *z* = 4.74, *p* < 0.001, *R*^2^ for entire model = 50.60%] than men (*b* = 0.30, *z* = 4.64, *p* < 0.001, *R*^2^ for entire model = 42.2%). However, an invariance test indicated that the model fit was statistically equivalent when this path was forced to be the same for men and women, χ^2^ difference (1) = 2.42, *p* = 0.12. The low number of women in the sample (*n* = 121) relative to men (*n* = 395) may have made it difficult to detect a significant interaction.

**FIGURE 4:**
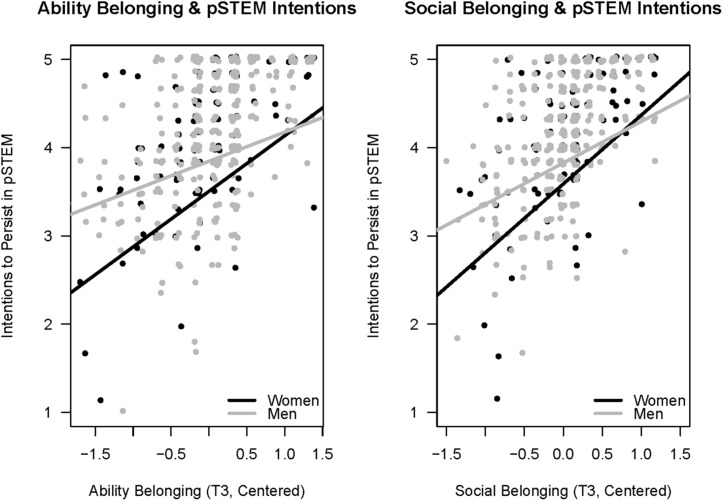
Intentions to persist as a function of belonging (ability on the left, social on the right), by gender. Lines depict partial relationships after controlling for professor, academic year, prior and ongoing performance, baseline belonging, as well as self-efficacy and identification at Time 1 and 3. Although the relationships are significant for both men and women, they are significantly stronger for women in the case of ability belonging.

Social belonging showed the same pattern of results, but here, the gender difference was significant (see Figure 3), and the relationship between social belonging and intentions to persist was roughly twice the size for women than for men. Model fit was significantly worse when the path between end-of-semester social belonging and intentions to persist in pSTEM was forced to be equivalent for men and women. This indicated that the path between end-of-semester social belonging and pSTEM persistence was significantly stronger for women (*b* = 0.77, *z* = 5.61, *p* < 0.001, *R*^2^ = 54.20%) than for men (*b* = 0.40, *z* = 5.17, *p* < 0.001, *R*^2^ = 46.10%), χ^2^ difference (1) = 5.30, *p* = 0.02. Figure 4 shows the relation between ability belonging (left panel) and social belonging (right panel) and intentions to persist separately for women and men. As can be seen, the pattern of results is similar, with both ability and social belonging more related to women’s intentions to persist in pSTEM than men’s intentions to persist, although important for both genders.

### Does Self-Efficacy or Identification Show a Gendered Relationship With Persistence?

For completeness, we also tested whether end-of-semester self-efficacy and identification were differentially related to intentions to persist in pSTEM for women and men. For both self-efficacy and identification, two invariance tests were conducted—for each of the models depicted in Figures 2, 3, we compared a model estimated separately by gender to a model in which the path between Time 3 self-efficacy or identification was constrained to be equivalent for men and women (i.e., one invariance test was based on a model controlling for ability belonging, and the other based on the model controlling for social belonging). In the context of controlling for either ability belonging or social belonging, the relationship between self-efficacy and intentions was statistically equivalent for men and women: χ^2^ differences (1) < 2.43, *p*s > 0.12. Similarly for identification, the relationship between end-of-semester identification and intentions to persist did not depend on gender, χ^2^ differences (1) < 0.04, *p*s > 0.84.

## Discussion

The current research expands upon prior work in two key ways. First, we examined the influence of both social and ability belonging on pSTEM persistence. Importantly for women and men alike, subjective ASPs of one’s social belonging, ability belonging, and identification each were uniquely related to intentions to persist in pSTEM, even after controlling for prior and ongoing performance. Indeed, models that included these ASPs predicted far more variation in intentions to persist in pSTEM than models that only included academic preparation. This has important implications for universities and pSTEM programs; although they cannot easily intervene to increase students’ math and science preparation, they can certainly aim to foster more welcoming pSTEM environments that normalize academic struggle.

Second, it is the first research we are aware of to examine belonging to a more broadly defined group (i.e., pSTEM) and demonstrate a link between pSTEM belonging and pSTEM persistence. In contrast, most prior research examines belonging to a particular classroom or particular pSTEM field (e.g., mathematics; Good et al., 2012). Our results indicate that students have an experience beyond their individual classrooms and majors and can reflect and report on their ASPs regarding overall “pSTEM culture.” This suggests that it may be wise to consider what kind of culture the institution is fostering at a broad level, not just within individual departments. The results also have implications for understanding the gender gap in pSTEM. Even among a selective group of women—within a sample of pSTEM majors who are highly identified with STEM, who have promising high-school GPAs, and many of whom had gained admission to a selective engineering college—women reported feeling more out of step intellectually with their peers than did men. Women also expressed less confidence that they could succeed on tasks important for academic success (i.e., they reported lower self-efficacy). At the same time, women were even more likely to care about their pSTEM ability and performance, reporting greater pSTEM identification than men. Notably, this combination of stronger identification and low self-efficacy may make women particularly susceptible to stereotype threat (Schmader, 2002).

Although women reported lower pSTEM ability belonging than men, no gender difference emerged for pSTEM social belonging. The lack of a gender difference in social belonging was surprising given recent research findings that women in computing felt lower social belonging in their major and that women across multiple pSTEM majors expressed lower social belonging in a physics class (Lewis et al., 2017). One reason we may not replicate this research is that the level of measurement is different. Whereas the aforementioned research assessed belonging in a particular major or course, we assessed belonging at the broader pSTEM level, and research shows that the level at which belonging is measured is important (Freeman et al., 2007). That different patterns emerged for social and ability belonging further bolsters the importance of assessing these separate subcomponents of belonging in future research, as well differentiating between belonging in the specific classroom versus the broader field overall (pSTEM).

Consistent with past research, social belonging was strongly related to intentions to persist in pSTEM for both men and women, over and above self-efficacy and identification. In line with the vulnerability hypothesis, and of particular importance to explaining gender disparities in pSTEM, we found that social belonging at the end of the semester was more tightly related to women’s intentions to persist than men’s intentions to persist, even after accounting for powerful covariates. This replicates prior research showing that social belonging is particularly important to women’s pSTEM persistence (Lewis et al., 2017). This study allowed us to test whether ability belonging was also particularly important for women’s intentions to persist; although the correlation between ability belonging and intentions to persist in pSTEM was directionally stronger for women than men, it was not significantly different. Additionally, although there were mean gender gaps in identification and self-efficacy (with women expressing greater identification but lower self-efficacy), their relationship with intentions to persist in pSTEM was the same for men and women.

This research reinforces the critical role of social belonging in pSTEM pursuits for women and suggests that even when men and women report the same level of social belonging, this level may nevertheless be inadequate for women in terms of translating into actual persistence. A greater sensitivity to belonging experiences suggests that even if women experience the same average level of belonging as men, this may still not be “good enough” for women to be convinced that they belong. Women in pSTEM whose social or ability belonging needs go unmet may consider pursuing other fields where they anticipate experiencing greater social (Murphy and Zirkel, 2015) and ability belonging (Smith et al., 2013)—likely fields where there are more women (Thoman et al., 2014). Furthermore, understanding the extent to which both social and ability belonging matter for women more than men is important for informing interventions aimed at retaining more women in pSTEM. Whereas some interventions focus on bolstering social belonging (Walton et al., 2015), others focus on increasing ability belonging, typically by normalizing hard work (Smith et al., 2013) or the experience of academic struggle (Lin-Siegler et al., 2016).

### Limitations

Like most survey research, students opted to partake in the survey, and we cannot know whether results would differ if non-responders were included. Furthermore, Little’s (1988) MCAR test indicated that data were not missing completely at random. Given the longitudinal nature of the data, it is not surprising that not all subjects provided full data at each time point, and further, we handled missing data by using analytical techniques robust to missing data (i.e., FIML), even if data were not missing at random. Although we drew students from a total of six different pSTEM professors teaching two different courses, these students do not represent all pSTEM students at the university or at other universities. This research was correlational in nature, and thus, we cannot draw causal inferences about the relationship between belonging and intentions to persist. Future research should investigate whether, for example, interventions aimed at improving social and ability belonging may be particularly beneficial to women in pSTEM relative to men. We also measured intentions to persist in pSTEM pursuits rather than actual persistence. However, intentions measured at the same level of specificity as the behavior of interest are widely viewed as the most proximal predictor of actual behavior (Ajzen, 1985, 1987, 1991, 2011). Intentions are, therefore, often used to assess educational outcomes (e.g., Tinto, 1997; Murphy et al., 2007; Good et al., 2012; Lewis et al., 2017; Ito and McPherson, 2018).

### Future Research and Implications for Educational Practices

That qualified men and women who are entirely capable of success in pSTEM fields may nevertheless drop out due to feeling as though they do not “fit” socially or intellectually is a waste of intellectual talent. Attracting and retaining more women in pSTEM would not only supply a deficient workforce but also better address the needs of a diverse population (Blickenstaff, 2005) and potentially enhance the innovation, creativity, and quality of science produced (Hill et al., 2010; Hoever et al., 2012; Nielsen et al., 2017). It is our hope that future research examines how to boost each of these distinct types of belonging. For social belonging, interventions could entail creating inclusive environments that affirm women’s sense of social connection with peers. Such environments may strategically place women with female role models (Dennehy and Dasgupta, 2017), remove reminders of masculine stereotypes or culture (Cheryan et al., 2009), or attempt to place more than one woman in small work groups (Dasgupta et al., 2015; Grover et al., 2017). Regarding ability belonging, pSTEM environments should attempt to emphasize effort and hard work over brilliance and innate talent (Smith et al., 2013; Lin-Siegler et al., 2016). Messages that normalize the struggle and journey to find social and ability belonging—particularly among dominant group members—would likely also benefit students who are questioning whether their experience is “normal” (Walton et al., 2015). Notably, this approach may be in direct opposition to the competitive “weed-out” cultures that have been described as commonplace in introductory pSTEM courses (Seymour and Hewitt, 1997; Shapiro and Sax, 2011).

Research shows that anticipated belonging plays a key role in decisions about whether or not to pursue pSTEM (Cheryan et al., 2009) and that it may be a more important criterion for women’s pursuit of a field than men’s (McPherson et al., 2018a). This suggests that future research is needed to address the relationship between belonging and attraction to pSTEM fields, and to examine whether these relationships also depend on gender. For example, perhaps anticipated belonging in pSTEM is not only lower among women than men but also consistent with women’s greater focus on communal goals (Diekman et al., 2010), women may also weigh anticipated belonging more than men when selecting a major or a career (McPherson et al., 2018a).

On the theoretical level, it would also be interesting in future research to further consider the relation of different aspects of fit. Schmader and Sedikides (2018) have recently suggested that multiple aspects of fit all contribute to a sense of authenticity, a gestalt feeling of having one’s identity align with the environment, suggesting that social and ability belonging may relate to a superordinate construct of authenticity or general belonging.

Although the present research focuses on the greater attrition of women than men from pSTEM fields, it is important to keep in mind that the gender differences observed here were not of kind but of degree—social and ability belonging were related to pSTEM persistence for women and men alike, suggesting that interventions aimed at boosting either of these should benefit both genders. It is becoming increasingly clear that retaining more talented men and women within pSTEM fields will require creating socially and intellectually welcoming environments in which students feel as though they not only are socially accepted by their peers but also have the same intellectual capacity as their peers.

## Data Availability Statement

The datasets for this article are not publicly available, because they contain educational records. Requests to access the datasets should be directed to TI at tiffany.ito@colorado.edu.

## Ethics Statement

The studies involving human participants were reviewed and approved by CU Boulder Institutional Review Board. The patients/participants provided their written informed consent to participate in this study.

## Author Contributions

TI and KL designed and implemented the study. KL oversaw the data collection. SB and KL performed the data analyses. SB, TI, and KL wrote the manuscript.

## Conflict of Interest

The authors declare that the research was conducted in the absence of any commercial or financial relationships that could be construed as a potential conflict of interest.

## Footnotes

1 There is overlap between social belonging and the social dimension of the self-concept (e.g., Markus and Wurf, 1987; Bracken, 1996). However, because some views of the social dimension of the self-concept focus on perceptions of one’s social skills and competence (e.g., Bracken, 1992), whereas we are interested in subjective perceptions of acceptance, we prefer the term *belonging*.
