# Supplementary material for: The Role of Social and Ability Belonging in Men’s and Women’s pSTEM Persistence
Source: Front Psychol. 2019 Oct 31;10:2386. doi: 10.3389/fpsyg.2019.02386 (PMC6834781; doi:10.3389/fpsyg.2019.02386)
Supplement: Supplementary file 1 [file Table_1.docx]

The Role of Social and Ability Belonging in Men and Women’s pSTEM Persistence

Supplementary Material

**1 About the Calculus and Physics Courses**

Courses consisted of three, 50-minute lectures and one recitation section per week over the course of the semester. According to university records, each physics section had between 340-342 students, and each calculus section had between 106-145 students. According to the course catalogue, the physics course covered kinematics, dynamics, momentum of particles and rigid bodies, work and energy, gravitation, simple harmonic motion, and introduction to thermodynamics. The calculus course covered analytical geometry and calculus including limits, rates of change of functions, derivatives and integrals of algebraic and transcendental functions, applications of differentiations and integration.

**2 Qualifying pSTEM Majors for Inclusion in the Sample**

Applied Mathematics

Architectural Engineering

Aerospace Engineering Sciences

Astronomy

Atmospheric & Oceanic Science

Biochemistry

Chemistry

Chemical & Biological Engineering

Chemical Engineering

Engineering

Computer Science

Civil Engineering

Electrical and Computer Engineering

Electrical Engineering

Engineering Honors

Engineering – Open Option

Environmental Engineering

General Engineering

Engineering Physics

Geology

Mathematics

Mechanical Engineering

Physics

**3 Additional Measures in the Surveys not Relevant to Present Analyses**

In addition to the items reported in the main text, the following constructs were measured in the study, with same items indicated for each.

1. Course-level social belonging (e.g., I feel like I belong in [course name].)
2. Course-level ability belonging (e.g., I sometimes feel like other students in [course name] have skills that I don’t have.)
3. Course-level identification (e.g., It is important to me that I am good at physics.)
4. Course-specific self-efficacy (e.g., Please rate how confident you are that you can do each of the following things in this physics [or calculus] course…Complete homework assignments by myself.)
5. Entity theory of intelligence (e.g., People have a certain amount of intelligence, and you really can’t do much to change it.)
6. Gender-science stereotypes (e.g., According to my own personal beliefs, I expect men to generally do better in physics than women.)
7. Support for your major from close others (e.g., How supportive is each of the following person(s) of your choice of/major? Mother)
8. General social connection (e.g., I enjoy personal and mutual conversations with family members or friends.)
9. Professor support (e.g., The instructor gives good examples of the concepts.)
10. Recitation TA support (e.g., The TA gives good examples of the concepts.)
11. University STEM support (e.g., My instructors at [university name] have been interested in my progress in math and science.)
12. Liking for/comfort with STEM professors (e.g., How much do you like your math and science professors?)
13. Liking for/comfort with STEM students (e.g., How much do you like other students in your math and science classes?)
14. Perceived environmental entity theory (e.g., My [course name] professor believes that people have a certain amount of intelligence, and you really can’t do much to change it.)
15. Perceived environmental stereotypes (e.g., Students in [course name] find it hard to believe that a female could be a genius in physics.)
16. Belonging at the university (e.g., I feel like I belong at [university name].)
17. Course utility (e.g., I think about the physics [or calculus] I experience in everyday life.
18. Intentions to Persist at [university name] (e.g., I have no doubt that I will graduate with a degree from [university name].)
19. Potential (e.g., Please rate your potential to succeed in STEM relative to your classmates.)
20. Attendance (e.g., What percentage of lecture sessions did you attend?)

**4 Change in Social and Ability Belonging over the Semester**

Given the longitudinal nature of the dataset, four path models separately examined change in ability belonging, social belonging, self-efficacy, and identification from the beginning to the end of the semester by subtracting each student’s Time 1 score from their Time 3 score (e.g., Time 3 social belonging – Time 1 social belonging). For any variable where the participant had Time 2 data but not Time 3 data, the difference was defined as that between Time 2 and Time 1 (results were the same if these participants were dropped from analyses). Thus, the intercept of these models tests whether the academic self-perception changed over the course of the semester, controlling for all predictors. Each change was then regressed onto gender, academic year, high school GPA, standardized test scores (SAT and ACT standardized and averaged), and professor (5 orthogonal, contrast-coded predictors).

As shown in Supplemental Table 1, identification (unstandardized beta = -.37, *z* = -4.59, *p* < .001) and self-efficacy (unstandardized beta = -.21, *z* = -2.70, *p* = .007) dropped over the course of the semester, but social and ability belonging remained stable. Overall, demographics (gender, academic year) and prior performance (high school GPA, standardized test scores) explained little variation in changes over the semester, as evidenced by low *R^2^*, percent variation in the change explain by the predictors. Academic year moderated the drop in self-efficacy, such that students who were academically more advanced experienced less of a drop. The only other significant differences occurred for one of the professor codes, suggesting that there were some professor differences in the degree to which the academic self-perceptions changed over the semester. The only change in academic self-perceptions that depended on gender was social belonging, unstandardized beta = .06, *z* =1.99, *p* = .05. Simple effects revealed that for women, belonging directionally increased throughout the semester, *p* = .11, whereas for men, it directionally decreased, *p* = .96, although neither slope was significant.

**5 Statistical Tests of Multicollinearity**

To test for multicollinearity, we examined the variance inflation factors (VIFs), which indicate the extent to which variation in the model is inflated by the presence of correlation among predictor variables, in a model regressing intentions to persist in pSTEM onto all four academic self-perceptions at Time 1 and Time 3 (each of which was mean-centered), high-school GPA (mean-centered), standardized test performance, pSTEM GPA (centered) gender (contrast-coded), academic year (contrast-coded) and professor (5 orthogonal contrast-coded predictors to account for nesting due to six different professors).

This model indicated that inclusion of both ability and social belonging at Time 3 increased the variance inflation factor by nearly 3, indicating potentially problematic redundancy (Wooldridge, 2013). Specifically, the VIF for social belonging at Time 3 was 2.93 (See Table 2). One remedy to this issue would be to treat ability and social belonging as a single variable, averaging them together. However,ability and social belonging are viewed as related yet theoretically distinct (see Lewis & Hodges, 2015). Furthermore, as described in the main text, comparisons of two nested confirmatory factor analysis (CFA) models showed that model fit was significantly better when ability and social belonging at each time point are treated as separate factors.

A second remedy, which we ultimately adopted, was to examine the relationships between ability and social belonging and intentions to persist in pSTEM in two separate models in order to remove the issue of multicollinearity.

Supplemental Table 1

*Predictors of Change in Academic Self-perceptions over Time*

| *Outcomes* | Ability Belonging | Social Belonging | Self-efficacy | Identification |
| --- | --- | --- | --- | --- |
| *Predictors* |  |  |  |  |
| Intercept (Change) | -0.01 | 0.06 | -0.12** | -0.19*** |
| Gender | -0.03 | 0.06 | 0.00 | 0.00 |
| Academic Year | -0.08 | -0.08 | 0.21* | -0.09 |
| High school GPA | 0.01 | -0.05 | 0.07 | 0.00 |
| SAT and ACT math & science | -0.04 | 0.07* | 0.07+ | 0.00 |
| Prof Code 1 (Physics vs. Calculus) | 0.01 | 0.00 | -0.02 | 0.01 |
| Prof Code 2 | 0.03 | 0.00 | -0.02 | -0.02 |
| Prof Code 3 | -0.02 | -0.02 | 0.02 | -0.01 |
| Prof Code 4 | 0.05 | 0.04 | -0.02 | -0.05 |
| Prof Code 5 | 0.13* | -0.09* | -0.11* | -0.06 |
| *R^2^* | 3.00% | 3.70% | 4.70% | 1.80% |

*Note*. ****p* < .001; ***p* < .01; **p* < .05, +*p* < .10. ASP = academic self-perceptions. Prof = Professor. For each ASP, the outcome variable is Time 3 – Time 1, and therefore the intercept represents change on average across the other predictors. Models were estimated using FIML, thus they are based on the entire sample (*N* = 516). All continuous predictors were mean centered, and categorical predictors were orthogonally contrast-coded. Significant effects are bolded. Estimates are unstandardized.

Supplemental Table 2

*Variance Inflation Factors (VIF) in a Regression Model Predicting Intentions to Persist in pSTEM*

| Predictors | VIF |
| --- | --- |
| Gender | 1.27 |
| Academic Year | 1.18 |
| High school GPA | 1.30 |
| SAT and ACT math & science | 1.58 |
| pSTEM GPA | 1.72 |
| Prof Code 1 (Physics vs. Calculus) | 1.37 |
| Prof Code 2 | 1.14 |
| Prof Code 3 | 1.19 |
| Prof Code 4 | 1.53 |
| Prof Code 5 | 1.08 |
| Ability Belonging (Time 3) | 2.21 |
| Ability Belonging (Time 1) | 2.38 |
| Social Belonging (Time 3) | 2.93 |
| Social Belonging (Time 1) | 2.50 |
| Self-efficacy (Time 3) | 1.85 |
| Self-efficacy (Time 1) | 1.96 |
| Identification (Time 3) | 1.82 |
| Identification (Time 1) | 1.71 |

Supplemental Table 3

*Parameter Estimates Social and Ability Belonging – Intentions to Persist Models*

| *Predictors* | No ASP | Add Belonging | Add Other ASP |
| --- | --- | --- | --- |
| Gender | 0.02 | -0.01 | -0.01 |
| Academic Year | -0.17 | -0.10 | -0.08 |
| High school GPA | **-0.36**** | **-0.36***** | **-0.31**** |
| SAT and ACT math & science | 0.04 | -0.01 | 0.00 |
| pSTEM GPA | **0.37***** | **0.15*** | **0.11^+^** |
| Prof Code 1 (Physics vs. Calculus) | 0.01 | 0.01 | 0.00 |
| Prof Code 2 | -0.02 | -0.03 | -0.03 |
| Prof Code 3 | -0.02 | -0.04 | -0.02 |
| Prof Code 4 | -0.01 | 0.00 | 0.01 |
| Prof Code 5 | -0.08 | -0.06 | -0.03 |
| Social Belonging (Time 3) |  | **0.46***** | **0.33***** |
| Social Belonging (Time 1) |  | 0.13^+^ | 0.09 |
| Ability Belonging (Time 3) | - | **0.19**** | **0.19**** |
| Ability Belonging (Time 1) | - | 0.02 | .05 |
| Identification (Time 3) | - | - | **0.31***** |
| Identification (Time 1) | - | - | 0.05 |
| Self-efficacy (Time 3) | - | - | 0.04 |
| Self-efficacy (Time 1) | - | - | 0.05 |
| *R^2^* | 15.60% | 39.70% | 46.10% |

*Note.* ****p* < .001, ***p* < .01, **p* < .05, ^+^*p* < .10. Prof = Professor. ASP = academic self-perceptions. Models were estimated using FIML, thus they are based on the entire sample (*N* = 516). All continuous predictors were mean centered, and categorical predictors were orthogonally contrast-coded. Significant effects are bolded. Estimates are unstandardized.
